# Associations of Dietary Intakes with Gynecological Cancers: Findings from a Cross-Sectional Study

**DOI:** 10.3390/nu14235026

**Published:** 2022-11-25

**Authors:** Guixian Zhu, Zengbin Li, Liqiong Tang, Mingwang Shen, Zhangjian Zhou, Yuhang Wei, Yang Zhao, Shuheng Bai, Lingqin Song

**Affiliations:** 1 Department of Oncology, The Second Affiliated Hospital, Xi’an Jiaotong University, No. 157, Xiwu Road, Xi’an 710004, China; 2 China-Australia Joint Research Center for Infectious Diseases, School of Public Health, Xi’an Jiaotong University Health Science Center, Xi’an 710061, China; 3 Medical School, Xi’an Jiaotong University, Xi’an 710061, China

**Keywords:** gynecological cancers, cervical cancer, ovarian cancer, endometrial cancer, dietary factors, nutrients, NHANES

## Abstract

Background: Gynecological cancers, including cervical cancer, ovarian cancer and endometrial cancer are leading causes of cancer-related death in women worldwide. Diet plays an important role in cancer development, which is widely accepted. However, the associations between dietary intakes and gynecological cancers remain unclear. Methods: A total of 12,437 women aged over 20 years from the National Health and Nutrition Examination Survey (NHANES), conducted from 2007–2016, were included in this study. The relationships between 30 dietary factors (4 macronutrients, 15 vitamins, 9 minerals, caffeine and alcohol) and gynecological cancers were assessed. Results: We observed negative correlations of intakes of phosphorus (odds ratio (OR), 95% confidence interval (CI); 0.998 (0.996, 0.999), *p* = 0.002) with cervical cancer, and intakes of vitamin B12 (0.812 (0.714, 0.925), *p* = 0.002), phosphorus (0.997 (0.996, 0.999), *p* < 0.001) and alcohol (0.971 (0.950, 0.992), *p* = 0.009) with endometrial cancer. The data showed positive associations of intake of caffeine (1.002 (1.001, 1.003), *p* = 0.003) with cervical cancer, and intake of copper (2.754 (1.313, 5.778), *p* = 0.009) with endometrial cancer. In addition, we found potential negative correlations between intake of vitamin B1 (*p* = 0.025) and cervical cancer; zinc (*p* = 0.048) and ovarian cancer; and potassium (*p* = 0.032) and endometrial cancer. Potential positive associations were found between intake of calcium and cervical cancer (*p* = 0.026) and endometrial cancer (*p* = 0.034), and between sodium (*p* = 0.042) and endometrial cancer. Intakes of protein, total sugars, total fat, cholesterol, vitamin A, alpha-carotene, beta-carotene, beta-cryptoxanthin, lycopene, vitamin B2, niacin, vitamin B6, food folate, vitamin C, vitamin D, vitamin E, vitamin K, magnesium, iron and selenium showed no relationship with gynecological cancers (*p* > 0.05). Conclusions: Specific dietary factors were associated with gynecological cancers. More epidemiological studies are needed to validate our results.

## 1. Introduction

The incidence of gynecological cancers is growing worldwide and poses a serious public health problem [1]. Gynecological cancers, including cervical cancer, ovarian cancer and endometrial cancer, are leading causes of cancer-related death in women globally [2]. Ovarian cancer ranks fifth and endometrial cancer ranks sixth in cancer-related mortality in the United States (U.S.), separately [3]. Although significant progress has been made toward early detection and treatment, cervical cancer, ovarian cancer and endometrial cancer are usually detected at a late stage and have a poor prognosis [4,5]. Thirty to sixty percent of cancers are closely related to dietary factors in the developed countries [6]. The associations between dietary intakes and cancers, such as colorectal cancer [7], breast cancer [8], prostate cancer [9] and lung cancer [10], have been studied extensively so far. Lifestyle changes such as dietary transformation are typically proposed to prevent the development of gynecological cancers, and to mitigate severity once the disease has developed [11].

There has been renewed interest in relationships between dietary factors and gynecological cancer risk [12,13,14]. Antioxidants, such as some vitamins, carotenoids, vegetables and fruits, exhibit different effects on the natural history of gynecological cancers [15,16,17]. However, results on the relationships between dietary intakes and gynecological cancers remain limited and inconclusive [18,19,20]. For instance, recent studies showed that alcohol intake has variably shown positive (odds ratio (OR), 95% confidence interval (CI), 1.6 (1.2, 2.2)) [21], inverse (risk ratio (RR), 95%CI, 0.81 (0.68, 0.96)) [22] or null association (RR, 95%CI, 1.04 (0.88, 1.22)) [23] with endometrial cancer. Moreover, previous studies only focused on a single dietary factor, ignoring the complexity and multitude of dietary variables, so that reported results on the associations between dietary intakes and gynecological cancers have been one-sided [14,24,25,26,27]. Modifications in one dietary characteristic usually led to compensatory changes in other dietary characteristics. Instead, focusing on food groups or dietary patterns could avoid the collinearity of dietary variables, and avoid finding chance associations due to analyses with multiple individual nutrients as the exposure [28,29]. Therefore, by considering multiple dietary factors, a comprehensive perspective of associations of diet with disease process can be provided [30].

The National Health and Nutrition Examination Survey (NHANES), as a consecutive design, was committed to measuring the health and nutrients status of non-institutionalized U.S. civilians [31]. This is the first study, as far as we know, to comprehensively investigate the relationships between dietary intakes and gynecological cancers using NHANES data. This study aimed to examine the associations between dietary intakes and gynecological cancers with a representative sample of Americans. In-depth understanding of the associations between dietary intakes and gynecological cancers provides a reference for preventing the occurrence and development of gynecological cancers.

## 2. Methods

### 2.1. Study Design and Population

Samples for this study were extracted from the NHANES, a multistage stratified composite design survey of a representative selection of the noninstitutionalized U.S. population. Participants were interviewed at home, followed by various clinical and laboratory examinations performed in a mobile examination center (MEC). We combined 5 consecutive NHANES surveys, 2007–2008, 2009–2010, 2011–2012, 2013–2014 and 2015–2016, into a single analytic sample. A total of 12,437 women aged over 20 years who were interviewed regarding their dietary intakes and medical conditions were included.

### 2.2. Outcomes

The primary outcomes were the diagnoses of gynecological cancers. Cancer types were defined using items on the Medical Status Questionnaire: “Have you ever been told by a doctor or other health professional that you had cancer or malignancy?” and “What kind of cancer was it?”. Answerers which indicated only cervical cancer, ovarian cancer and endometrial cancer were classified as outcome variables.

### 2.3. Dietary Intakes

Trained interviewers conducted two consecutive 24-h dietary recalls to assess total dietary intakes by comprehensive reference in the NHANES. The first was conducted face-to-face at the MEC examination and the second was collected by telephone after 3–10 days. Dietary intakes were calculated from the average of data from two dietary recalls (if available); otherwise, single dietary recall data were used.

We included 30 dietary factors from the dietary questionnaire of the NHANES database. These factors encompassed 4 macronutrients, 15 vitamins and 9 minerals—including protein (g), total sugars (g), total fat (g), cholesterol (mg), vitamin A (vitamin A, RAE) (μg), alpha-carotene (μg), beta-carotene (μg), beta-cryptoxanthin (μg), lycopene (μg), vitamin B1 (thiamin) (mg), vitamin B2 (riboflavin) (mg), niacin (mg), vitamin B6 (mg), food folate (μg), vitamin B12 (μg), vitamin C (μg), vitamin D (D2 + D3) (μg), vitamin E (vitamin E as alpha-tocopherol) (mg), vitamin K (μg), calcium (mg), phosphorus (mg), magnesium (mg), iron (mg), zinc (mg), copper (mg), sodium (mg), potassium (mg) and selenium (mcg)—as well as caffeine (mg) and alcohol (g).

### 2.4. Covariates

Covariates were previously identified as potential prognostic factors in recent literature [32,33,34]. We assessed demographic covariates including age, body mass index (BMI), poverty-to-income ratio (PIR), energy intake (expressed in kcal), education level, race, work activity and recreational activity [11,34,35,36].

### 2.5. Statistical Analysis

Descriptive analysis was used for exploring demographic characteristics of included participants. Considering that all continuous variables did not obey normal distribution, the median with interquartile range (IQR) was used to describe continuous variables. Significance difference between the two groups was appraised by Wilcoxon rank-sum test. Frequency and percent were used to describe categorical variables. The distribution of categorical variables was appropriately compared by chi-squared test.

Outcome variables were imbalanced due to the relatively low incidence of gynecological cancers. Thus the “ROSE” package in R software was used to correct the imbalanced data [37]. Furthermore, we considered the stratified, multi-stage probabilistic sampling approach of NHANES, using the “survey” package to adjust complex sampling weights for dietary analysis. Five cycles of continuous NHANES data from 2007–2016 were included. Two-year cycle weights were divided by 5 to reflect 10 survey years. Estimates and standard errors were analyzed using sampling weights, stratification and clustering provided in the NHANES dataset.

Logistic regression was performed using the “glmnet” package in R software. For the logistic regression, self-reported gynecological cancers were included as the dependent variables. All statistical analyses were conducted using IBM SPSS (version 24.0.) and R (version 4.1.2.). The *p*-value was adjusted for multiple comparisons using the Bonferroni correction (Bonferroni: 0.05/3 = 0.0167). A value of *p* < 0.0167 was considered statistically significant and 0.0167 < *p* < 0.05 indicated potential association.

## 3. Results

### 3.1. Characteristics of Included Participants

**Figure 1:**
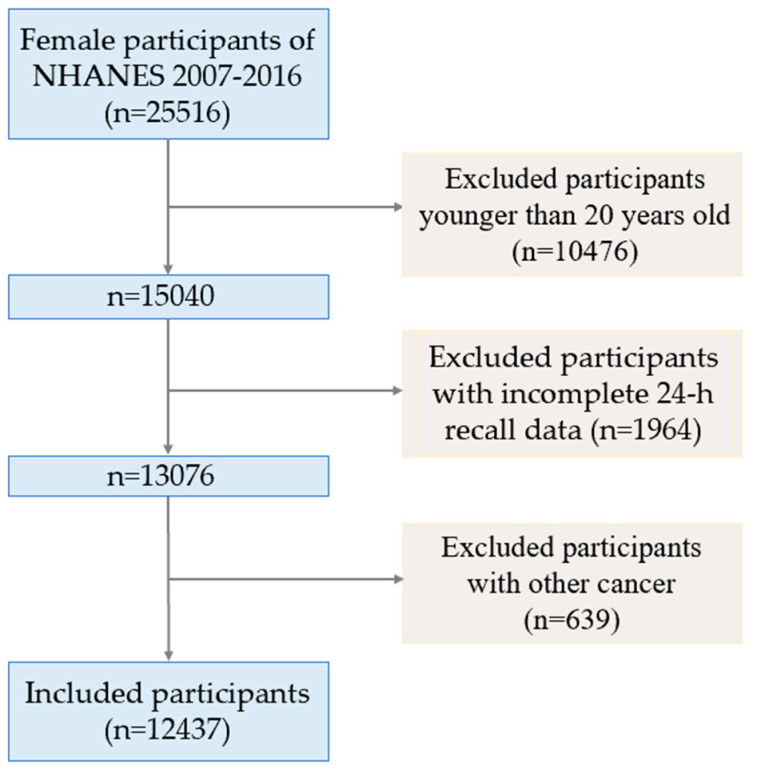
Flowchart of study participants.

The flow chart of participants is shown in Figure 1. Compared to the participants without cancers, those with cervical cancer had a lower PIR (*p* < 0.001), represented lower educational level (less than 9th grade, *p* = 0.009), tended to be non-Hispanic white (*p* < 0.001) and represented more work activity (*p* = 0.005); those with ovarian cancer were more likely to be older (*p* < 0.001), represented lower educational level (*p* = 0.010) and lower recreational activity (*p* = 0.025); and those with endometrial cancer were more likely to be older (*p* < 0.001), had a potentially lower PIR (*p* = 0.047), had higher BMI (*p* < 0.001), represented lower educational level (*p* < 0.001), tended to be non-Hispanic white (*p* = 0.012) and had lower recreational activity (*p* = 0.004). Characteristics of the included participants are summarized in Table 1.

### 3.2. Dietary Intakes and Gynecological Cancer Risk

Table 2 presents the dietary intakes of the included participants. Women suffering from cervical cancer took less alpha-carotene (*p* < 0.001), beta-carotene (*p* < 0.001), beta-cryptoxanthin (*p* = 0.001), vitamin B1 (*p* = 0.003), niacin (*p* = 0.009), vitamin B6 (*p* = 0.001), food folate (*p* = 0.007), vitamin C (*p* < 0.001), vitamin E (*p* = 0.015), vitamin K (*p* = 0.003) and iron (*p* = 0.003). In addition, women suffering from cervical cancer potentially took less protein (*p* = 0.048), vitamin A (*p* = 0.018) and copper (*p* = 0.048). Women suffering from ovarian cancer potentially took less zinc (*p* = 0.030) and selenium (*p* = 0.038).

Participants with cervical cancer tended to consume more caffeine (*p* < 0.001). Participants with ovarian cancer tended to consume more beta-cryptoxanthin (*p* = 0.011). Participants with endometrial cancer tended to consume more alcohol (*p* = 0.005). No statistical difference was observed in the other dietary factors with gynecological cancers (*p* > 0.05).

The correlations between dietary intakes and the gynecological cancers after adjustment for potential confounders are shown in Table 3. After adjusting for covariates, intake of phosphorus (OR, 95% CI; 0.998 (0.996, 0.999), *p* = 0.002) was negatively linked with cervical cancer; intakes of vitamin B12 (0.812 (0.714, 0.925), *p* = 0.002), phosphorus (0.997 (0.996, 0.999), *p* < 0.001) and alcohol (0.971 (0.950, 0.992), *p* = 0.009) were negatively linked with endometrial cancer. Intake of caffeine (1.002 (1.001, 1.003), *p* = 0.003) was positively associated with cervical cancer and intake of copper (2.754 (1.313, 5.778), *p* = 0.009) was positively associated with endometrial cancer. In addition, intake of vitamin B1 (*p* = 0.025) had a potential negative link with cervical cancer; intake of zinc (*p* = 0.048) had a potential negative link with ovarian cancer; and intake of potassium (*p* = 0.032) had a potential negative link with endometrial cancer. Intake of calcium (*p* = 0.026) had a potential positive association with cervical cancer and intakes of calcium (*p* = 0.034) and sodium (*p* = 0.042) had potential positive associations with endometrial cancer.

Intakes of protein (*p* = 0.266; *p* = 0.367; *p* = 0.064), total sugars (*p* = 0.468; *p* = 0.332; *p* = 0.386), total fat (*p* = 0.293; *p* = 0.469; *p* = 0.160), cholesterol (*p* = 0.533; *p* = 0.313; *p* = 0.477), vitamin A (*p* = 0.123; *p* = 0.151; *p* = 0.450), alpha-carotene (*p* = 0.981; *p* = 0.214; *p* = 0.253), beta-carotene (*p* = 0.213; *p* = 0.072; *p* = 0.460), beta-cryptoxanthin (*p* = 0.356; *p* = 0.505; *p* = 0.705), lycopene (*p* = 0.905; *p* = 0.194; *p* = 0.775), vitamin B2 (*p* = 0.182; *p* = 0.476; *p* = 0.237), niacin (*p* = 0.134; *p* = 0.749; *p* = 0.731), vitamin B6 (*p* = 0.146; *p* = 0.510; *p* = 0.212), food folate (*p* = 0.597; *p* = 0.484; *p* = 0.509), vitamin C (*p* = 0.639; *p* = 0.837; *p* = 0.993), vitamin D (*p* = 0.349; *p* = 0.595; *p* = 0.504), vitamin E (*p* = 0.392; *p* = 0.471; *p* = 0.715), vitamin K (*p* = 0.210; *p* = 0.455; *p* = 0.277), magnesium (*p* = 0.619; *p* = 0.832; *p* = 0.894), iron (*p* = 0.372; *p* = 0.683; *p* = 0.451) and selenium (*p* = 0.625; *p* = 0.674; *p* = 0.969) showed no relationship with cervical cancer, ovarian cancer and endometrial cancer, separately.

## 4. Discussion

In this cross-sectional study, we revealed the relationships between dietary intakes and gynecological cancers. Intakes of certain vitamins, minerals, caffeine and alcohol were positively associated with gynecological cancers. In addition, statistical diversities were represented between dietary factors and various types of gynecological cancers. As far as we know, it is the first study to comprehensively examine the relationships between dietary factors and gynecological cancers among the U.S. population using the NHANES database.

The prospect that intakes of certain vitamins might confer protection against cancer has received much attention during recent years [38,39,40]. Our study found that intake of vitamin B12 was negatively associated with endometrial cancer. However, epidemiological evidence exploring the link between vitamin B12 intake and endometrial cancer is limited, and the results are not consistent [41,42]. A study reported that no relationship between vitamin B12 consumption and endometrial cancer was observed [41]. In a prospective study, it was found that an initial increase in consumption of vitamin B12 did not enhance endometrial cancer risk, but the women in the highest consumption quintile had a significantly increased endometrial cancer risk compared to women in the lowest quintile [42]. In fact, such association was only observed in women with BMI ≥ 25 kg/m^2^ [42]. Our findings suggested that intake of vitamin B12 might have a negative relationship with endometrial cancer. It might be attributed to the potential mechanism whereby vitamin B12 deficiency could result in altered expression of cancer-related genes by reducing DNA synthesis, leading to cancer pathogenesis [43].

Whether dietary phosphorus affects the development of gynecological cancers is unclear [44]. Our findings suggested that phosphorus was protective against cervical cancer and endometrial cancer. Similar to our findings, recent studies have indicated that dietary phosphorus had a protective role in cervical intraepithelial neoplasia (CIN) and colorectal adenoma [45,46,47]. Combining both low calcium and high phosphorus might affect cancer development [48]. A statistical relationship between dietary ratio of calcium-to-phosphorus and CIN risk was reported in a previous case–control study [49]. However, these studies only focused on the dietetical calcium-to-phosphorus ratio and phosphorus inorganic salt status, not dietary phosphorus intake. The phosphorus intake reflected normal daily intake, but it might not embody an accurate level in cells [47]. The associations between phosphorus intake and gynecological cancers require further epidemiological studies to verify. In addition, we found that intake of copper might be positively associated with endometrial cancer. Elevated copper concentrations have been reported in many types of cancers, including gynecological cancers [50], breast cancer [51], lung cancer [52] and gastrointestinal cancer [53]. Relationships between copper and cancers have been widely accepted, as there is a requirement for higher levels of copper for cancer cells compared with non-dividing cells [54]. Copper appears to drive the estrogen-dependent cell proliferation [55,56,57].

We found that intakes of caffeine and alcohol were associated with gynecological cancers. Previous studies have demonstrated that intake of caffeine promoted development of specific cancers, such as esophageal adenocarcinoma [58], vulvar cancer [59] and head and neck squamous cell carcinomas [60]. A positive association between intake of caffeine and cervical cancer was observed in this study. It might be in part explained by the fact that caffeine, acting as an adenosine receptor, has been shown to contribute to carcinogenesis indirectly [61]. Our results observed that alcohol consumption was inversely linked with endometrial cancer even after accounting for multiple dietary factors. It has been raised that alcohol consumption might enhance cancer risk [62]. However, a recent large prospective cohort study among 68,067 women reported that, compared with women who did not drink, women who drank < 5 g/day of ethanol had a 22% reduction in risk of endometrial cancer [22]. When stratifying by alcohol consumption and BMI, alcohol consumption was associated with high risk of endometrial cancer in thin women (BMI < 25 kg/m^2^), whereas heavier women had a decreased risk [63,64]. The association between alcohol consumption and endometrial cancer needs more epidemiological studies to validate.

We also found potential links between several dietary factors and gynecological cancers. Our study indicated that intakes of vitamin B1, zinc and potassium had potential negative associations with cervical cancer, ovarian cancer and endometrial cancer, separately. Zhou et al. [65] reported that moderate intake of vitamin B1 could prevent human papilloma virus (HPV) infection, suggesting a resistant effect of vitamin B1 in cervical cancer. Previous studies reported that intakes of zinc and potassium were negatively associated with several cancers, such as breast cancer [66,67], lung cancer [36,68] and colorectal cancer [69,70]. Additionally, in a mendelian randomization analysis, women with high circulating zinc concentration had a lower risk of ovarian cancer [71]. Our results suggested that calcium intake had a potential positive relationship with cervical cancer and endometrial cancer. An epidemiological study showed a positive relationship between calcium and prostate cancer, even adjusting for race, dietary phosphorus and BMI [72]. We found that dietary sodium might have a potential positive association with endometrial cancer. It has been reported that low sodium intake might have benefits for human cancers [73]. Although the role of sodium intake in gynecological cancers has not been fully elucidated, sodium intake has been shown to have a positive association with renal cell cancer [74], lung cancer [68] and gastric cancer [75].

Some limitations of the study need to be acknowledged. First, self-reported 24-h dietary recall information was subjected to measurement bias due to large day-to-day changes in dietary intakes. The analysis relied on a single measurement of diet at a dietary recall interview, whereas dietary behaviors might change over time. Second, there might have been misclassifications in cancer status due to the self-reported nature of the survey. Participants might not have reported accurate information about their cancer history. Third, there were likely complex added effects and biological interactions between multiple nutrients and non-nutrients in the usual diet [76]. Although we adjusted for known covariates, residual confounding secondary unknown or uncontrolled factors such as estrogen use might have interfered with our results. Lastly, in this study, the dietary assessment was conducted after cancer diagnosis, hence the diet could have been modified after cancer treatment. Due to the cross-sectional nature of this investigation, causality was hard to elucidate in this investigation.

## 5. Conclusions

In conclusion, we found that intakes of copper and caffeine were positively related to certain gynecological cancers, while intakes of vitamin B12, phosphorus and alcohol were negatively related to them. In addition, intakes of vitamin B1, zinc, calcium, sodium and potassium had potential associations with gynecological cancers. Nevertheless, no significant correlations were observed between other dietary factors and gynecological cancers. Further epidemiological studies are required to elucidate our findings.

## Figures and Tables

**Table 1:** Characteristics of participants with and without self-reported cervical cancer, ovarian cancer and endometrial cancer, separately.

Variables	Women with Cervical Cancer (*n* = 162)	Women without Cervical Cancer (*n* = 12,275)	*p*-Value	Women with Ovarian Cancer (*n* = 66)	Women without Ovarian Cancer (*n* = 12,371)	*p*-Value	Women with Endometrial Cancer (*n* = 104)	Women without Endometrial Cancer (*n* = 12,333)	*p*-Value
Age (years), median (IQR) ^a^	46.50 (35.00, 58.25)	48.00 (34.00, 63.00)	0.401	62.50 (48.00, 71.00)	48.00 (34.00, 63.00)	<0.001 ***	60.00 (48.25, 69.00)	48.00 (34.00, 63.00)	<0.001 ***
Family Poverty-Income-Ratio, median (IQR) ^a^	1.63 (0.77, 2.69)	2.19 (1.08, 3.57)	<0.001 ***	1.73 (1.09, 3.33)	2.19 (1.08, 3.55)	0.584	2.05 (0.79, 2.57)	2.19 (1.08, 3.57)	0.047 *
BMI (kg/m^3^), median (IQR) ^a^	29.26 (24.20, 34.15)	28.60 (24.19, 33.70)	0.521	29.39 (26.50, 35.06)	28.60 (24.17, 33.70)	0.079	31.24 (26.08, 37.18)	28.60 (24.16, 33.70)	<0.001 ***
Energy (kcal), median (IQR) ^a^	1722.00 (1278.50, 2136.50)	1681.00 (1263.00, 2169.00)	0.658	1549.50 (1041.25, 2292.75)	1682.00 (1264.00, 2167.00)	0.325	1631.50 (1144.75, 1999.50)	1683.00 (1264.00, 2169.00)	0.153
Education, *n* (%) ^b^	-	-	0.009 **	-	-	0.010 *	-	-	<0.001 ***
Less than 9th grade	12 (7.4%)	1306 (10.6%)	-	14 (21.2%)	1304 (10.5%)	-	24 (23.1%)	1294 (10.5%)	-
9th−11th grade	36 (22.2%)	1731 (14.1%)	-	5 (7.6%)	1762 (14.2%)	-	19 (18.3%)	1748 (14.2%)	-
More than 12th grade	114 (70.4%)	9238 (75.3%)	-	47 (71.2%)	9305 (75.2%)	-	61 (58.7%)	9291 (75.3%)	-
Race, *n* (%) ^b^	-	-	<0.001***	-	-	0.791	-	-	0.012 *
Mexican American	15 (9.3%)	1960 (16.0%)	-	14 (21.2%)	1961 (15.9%)	-	16 (15.4%)	1959 (15.9%)	-
Other Hispanic	13 (8.0%)	1450 (11.8%)	-	7 (10.6%)	1456 (11.8%)	-	19 (18.3%)	1444 (11.7%)	-
Non-Hispanic White	114 (70.4%)	4909 (40.0%)	-	26 (39.4%)	4997 (40.4%)	-	51 (49.0%)	4972 (40.3%)	-
Non-Hispanic Black	12 (7.4%)	2713 (22.1%)	-	14 (21.2%)	2711 (21.9%)	-	12 (11.5%)	2713 (22.0%)	-
Other Race—including Multi-Racial	8 (4.9%)	1243 (10.1%)	-	5 (7.6%)	1246 (10.1%)	-	6 (5.8%)	1245 (10.1%)	-
Work activity, *n* (%) ^b^	-	-	0.005 **	-	-	0.950	-	-	0.145
Vigorous	30 (18.5%)	1347 (11.0%)	-	8 (12.1%)	1369 (11.1%)	-	7 (6.7%)	1370 (11.1%)	-
Moderate	39 (24.1%)	2736 (22.3%)	-	14 (21.2%)	2761 (22.3%)	-	30 (28.8%)	2745 (22.3%)	-
Other	93 (57.4%)	8192 (66.7%)	-	44 (66.7%)	8241 (66.6%)	-	67 (64.4%)	8218 (66.6%)	-
Recreational activity, *n* (%) ^b^	-	-	0.076	-	-	0.025 *	-	-	0.004 **
Vigorous	18 (11.1%)	2092 (17.0%)	-	5 (7.6%)	2105 (17.0%)	-	6 (5.8%)	2104 (17.1%)	-
Moderate	42 (25.9%)	3390 (27.6%)	-	14 (21.2%)	3418 (27.6%)	-	27 (26.0%)	3405 (27.6%)	-
Other	102 (63.0%)	6793 (55.3%)	-	47 (71.2%)	6848 (55.4%)	-	71 (68.3%)	6824 (55.3%)	-

^a^*p* value was tested by Wilcoxon rank-sum test; ^b^ *p* value was tested by Pearson chi-square test. * *p* < 0.05, ** *p* < 0.01, *** *p* < 0.001.

**Table 2:** Dietary intakes in women with and without self-reported cervical cancer, ovarian cancer and endometrial cancer, separately.

Variables Median (IQR)	Women with Cervical Cancer (*n* = 162)	Women without Cervical Cancer (*n* = 12,275)	*p*-Value	Women with Ovarian Cancer (*n* = 66)	Women without Ovarian Cancer (*n* = 12,371)	*p*-Value	Women with Endometrial Cancer (*n* = 104)	Women without Endometrial Cancer (*n* = 12,333)	*p*-Value
Protein (g)	58.53 (39.76, 79.42)	63.16 (45.76, 84.87)	0.048 *	56.77 (41.53, 79.73)	63.12 (45.76, 84.88)	0.086	62.50 (46.05, 82.96)	63.10 (45.72, 84.87)	0.708
Total sugars (g)	93.95 (52.88, 148.83)	86.85 (56.02,127.72)	0.166	77.21 (56.50, 143.16)	86.94 (55.97, 127.87)	0.807	84.36 (49.52, 129.05)	86.92 (56.03, 127.96)	0.601
Total fat (g)	62.00 (38.65, 87.61)	61.37 (41.07, 87.05)	0.888	56.65 (35.14, 84.04)	61.41 (41.08, 87.06)	0.166	56.41 (37.26, 82.12)	61.41 (41.09, 87.10)	0.144
Cholesterol (mg)	183.00 (97.00, 328.00)	187.00 (109.00, 323.00)	0.871	160.50 (100.75, 283.75)	187.00 (109.00, 324.00)	0.138	169.50 (93.25, 312.50)	187.00 (109.00, 323.00)	0.396
Vitamin A (μg)	362.00 (198.00, 612.25)	435.00 (240.00, 717.00)	0.018 *	464.00 (190.75, 690.25)	435.00 (239.00, 717.00)	0.719	490.00 (259.75, 819.25)	434.00 (239.00, 716.00)	0.203
Alpha-carotene (μg)	20.50 (1.00, 166.25)	48.00 (11.00, 270.00)	<0.001 ***	43.00 (10.75, 319.75)	47.00 (11.00, 269.00)	0.877	68.00 (18.25, 538.50)	47.00 (11.00, 267.00)	0.101
Beta-carotene (μg)	448.00 (137.75, 1294.50)	733.00 (267.00, 2376.00)	<0.001 ***	904.00 (148.00, 3051.00)	727.00 (264.00, 2351.00)	0.874	1061.50 (386.00, 2777.00)	726.00 (263.00, 2349.00)	0.089
Beta-cryptoxanthin (μg)	16.50 (3.75, 54.50)	27.00 (7.00, 90.00)	0.001 **	45.50 (15.50, 150.50)	26.00 (7.00, 90.00)	0.011 *	26.00 (10.00, 108.75)	26.00 (7.00, 90.00)	0.440
Lycopene (μg)	921.00 (0.00, 4612.75)	1422.00 (1.00, 4591.00)	0.153	1482.00 (0.00, 6221.25)	1417.00 (1.00, 4583.00)	0.796	1317.50 (5.25, 3957.75)	1418.00 (1.00, 4595.00)	0.844
Vitamin B1 (mg)	1.13 (0.80, 1.44)	1.24 (0.86, 1.68)	0.003 **	1.19 (0.76, 1.61)	1.24 (0.86, 1.68)	0.153	1.25 (0.86, 1.54)	1.24 (0.86, 1.68)	0.751
Vitamin B2 (mg)	1.61 (1.12, 2.34)	1.59 (1.12, 2.20)	0.429	1.52 (1.06, 2.04)	1.59 (1.12, 2.20)	0.270	1.55 (1.08, 2.11)	1.59 (1.12, 2.20)	0.517
Niacin (mg)	16.95 (12.34, 22.38)	18.70 (13.16, 25.71)	0.009 **	17.14 (12.45, 26.28)	18.67 (13.16, 25.66)	0.326	16.55 (12.52, 23.64)	18.68 (13.16, 25.69)	0.119
Vitamin B6 (mg)	1.25 (0.79, 1.88)	1.48 (1.00, 2.11)	0.001 **	1.27 (0.97, 2.01)	1.48 (1.00, 2.10)	0.303	1.38 (0.94, 1.90)	1.48 (1.00, 2.10)	0.230
Food folate (μg)	154.00 (92.50, 213.25)	165.00 (110.00, 240.00)	0.007 **	172.00 (115.00, 233.25)	165.00 (110.00, 240.00)	0.981	151.50 (104.50, 244.75)	165.00 (110.00, 240.00)	0.801
Vitamin B12 (μg)	3.05 (1.63, 5.02)	3.17 (1.78, 5.14)	0.586	2.59 (1.76, 4.08)	3.17 (1.78, 5.14)	0.185	3.24 (1.76, 5.07)	3.16 (1.78, 5.14)	0.914
Vitamin C (μg)	24.70 (9.15, 83.58)	51.30 (20.90, 107.50)	<0.001 ***	64.40 (24.93, 122.58)	51.00 (20.60, 107.30)	0.220	59.65 (25.73, 101.45)	50.90 (20.70, 107.40)	0.493
Vitamin D (μg)	2.70 (0.80, 4.73)	2.80 (1.10, 5.40)	0.240	3.05 (1.55, 5.23)	2.80 (1.10, 5.40)	0.623	3.10 (1.53, 5.45)	2.80 (1.10, 5.40)	0.485
Vitamin E (mg)	5.21 (3.14, 8.31)	5.96 (3.80, 9.00)	0.015 *	5.20 (3.20, 9.31)	5.95 (3.80, 9.00)	0.407	5.45 (3.70, 8.04)	5.96 (3.79, 9.00)	0.216
Vitamin K (μg)	51.50 (25.43, 88.73)	58.60 (32.50, 112.80)	0.003 **	60.35 (25.30, 115.55)	58.60 (32.40, 112.30)	0.518	54.80 (34.03, 106.63)	58.60 (32.30, 112.40)	0.563
Calcium (mg)	671.50 (412.75, 1111.25)	732.00 (479.00, 1054.00)	0.257	653.00 (459.75, 982.75)	732.00 (477.00, 1055.00)	0.332	742.50 (477.00, 1004.75)	731.00 (477.00, 1055.00)	0.946
Phosphorus (mg)	1022.00 (703.50, 1391.00)	1075.00 (783.00, 1429.00)	0.075	947.50 (660.50, 1313.25)	1075.00 (782.00, 1429.00)	0.140	968.50 (789.75, 1406.00)	1075.00 (782.00, 1429.00)	0.205
Magnesium (mg)	224.00 (145.75, 306.25)	239.00 (175.00, 320.00)	0.093	229.50 (162.75, 323.00)	239.00 (175.00, 320.00)	0.447	230.00 (189.25, 303.25)	239.00 (175.00, 320.00)	0.800
Iron (mg)	10.04 (7.12, 13.62)	11.22 (7.83, 15.60)	0.003 **	9.84 (6.77, 13.83)	11.20 (7.83, 15.60)	0.064	10.58 (7.77, 15.25)	11.20 (7.82, 15.59)	0.745
Zinc (mg)	7.70 (5.39, 11.27)	8.29 (5.76, 11.63)	0.221	7.43 (5.15, 9.38)	8.29 (5.76, 11.63)	0.030 *	8.47 (5.64, 11.39)	8.28 (5.76, 11.62)	0.820
Copper (mg)	0.95 (0.60, 1.30)	0.99 (0.71, 1.35)	0.048 *	0.90 (0.61, 1.33)	0.99 (0.71, 1.35)	0.153	1.03 (0.72, 1.39)	0.99 (0.71, 1.35)	0.638
Sodium (mg)	2579.00 (1807.50, 3561.75)	2729.00 (1955.00, 3676.00)	0.172	2359.00 (1701.50, 3648.25)	2726.00 (1954.00, 3674.00)	0.164	2735.50 (1836.75, 3607.75)	2725.00 (1952.50, 3675.50)	0.576
Potassium (mg)	2063.50 (1370.75, 2881.00)	2151.00 (1567.00, 2851.00)	0.125	2303.50 (1454.50, 2996.00)	2150.00 (1565.00, 2850.00)	0.773	2163.00 (1604.75, 2905.00)	2150.00 (1564.50, 2851.00)	0.866
Selenium (μg)	80.95 (51.45, 111.60)	86.50 (60.80, 118.90)	0.057	75.65 (51.35, 112.70)	86.50 (60.80, 118.90)	0.038 *	80.30 (56.63, 120.68)	86.50 (60.70, 118.80)	0.341
Caffeine (mg)	185.00 (44.50, 329.75)	83.00 (9.00, 180.00)	<0.001 ***	105.50 (22.50, 201.00)	83.00 (9.00, 183.00)	0.229	76.00 (6.50, 169.25)	84.00 (9.00, 183.00)	0.458
Alcohol (g)	0.00 (0.00, 0.00)	0.00 (0.00, 0.00)	0.711	0.00 (0.00, 0.00)	0.00 (0.00, 0.00)	0.894	0.00 (0.00, 0.00)	0.00 (0.00, 0.00)	0.005 **

*p* value was tested by Wilcoxon rank-sum test, median (IQR). * *p* < 0.05, ** *p* < 0.01, *** *p* < 0.001.

**Table 3:** ORs with 95% CIs of the associations between dietary intakes, cervical cancer, ovarian cancer and endometrial cancer, separately.

Variables	Cervical Cancer OR (CI)	*p*-Value	Ovarian Cancer OR (CI)	*p*-Value	Endometrial Cancer OR (CI)	*p*-Value
Protein (g)	1.013 (0.990, 1.037)	0.266	1.012 (0.986, 1.039)	0.367	1.026 (0.998, 1.053)	0.064
Total sugars (g)	1.003 (0.995, 1.010)	0.468	1.005 (0.995, 1.015)	0.332	1.005 (0.993, 1.017)	0.386
Total fat (g)	1.010 (0.992, 1.028)	0.293	1.009 (0.985, 1.033)	0.469	0.987 (0.968, 1.006)	0.160
Cholesterol (mg)	0.999 (0.998, 1.001)	0.533	0.999 (0.997,1.001)	0.313	1.001 (0.999, 1.002)	0.477
Vitamin A (μg)	0.999 (0.998, 1.000)	0.123	0.999 (0.998, 1.000)	0.151	1.000 (0.999, 1.001)	0.450
Alpha-carotene (μg)	1.000 (1.000, 1.000)	0.981	1.000 (1.000, 1.000)	0.214	1.000 (1.000, 1.000)	0.253
Beta-carotene (μg)	1.000 (1.000, 1.000)	0.213	1.000 (1.000, 1.000)	0.072	1.000 (1.000, 1.000)	0.460
Beta-cryptoxanthin (μg)	1.001 (0.999, 1.002)	0.356	1.000 (0.999, 1.001)	0.505	1.000 (0.999, 1.001)	0.705
Lycopene (μg)	1.000 (1.000, 1.000)	0.905	1.000 (1.000, 1.000)	0.194	1.000 (1.000, 1.000)	0.775
Vitamin B1 (mg)	0.518 (0.293, 0.916)	0.025 *	1.099 (0.678, 1.780)	0.695	0.565 (0.276, 1.155)	0.115
Vitamin B2 (mg)	1.362 (0.861, 2.154)	0.182	0.775 (0.378, 1.586)	0.476	1.465 (0.770, 2.785)	0.237
Niacin (mg)	0.965 (0.921, 1.012)	0.134	0.990 (0.930, 1.054)	0.749	1.011 (0.950, 1.075)	0.731
Vitamin B6 (mg)	1.408 (0.883, 2.246)	0.146	1.170 (0.726, 1.885)	0.510	1.232 (0.884, 1.717)	0.212
Food folate (μg)	0.999 (0.996, 1.002)	0.597	0.999 (0.996, 1.002)	0.484	1.001 (0.998, 1.003)	0.509
Vitamin B12 (μg)	1.067 (0.968, 1.177)	0.186	1.031 (0.942,1.129)	0.494	0.812 (0.714, 0.925)	0.002 **
Vitamin C (μg)	0.999 (0.995, 1.003)	0.639	1.001 (0.995, 1.006)	0.837	1.000 (0.996, 1.004)	0.993
Vitamin D (μg)	1.032 (0.966, 1.102)	0.349	1.015 (0.959, 1.075)	0.595	1.026 (0.949, 1.110)	0.504
Vitamin E (mg)	0.979 (0.932, 1.029)	0.392	0.979 (0.922, 1.039)	0.471	0.990 (0.938, 1.045)	0.715
Vitamin K (μg)	0.999 (0.997, 1.001)	0.210	0.999 (0.997, 1.001)	0.455	0.999 (0.997, 1.001)	0.277
Calcium (mg)	1.001 (1.000, 1.002)	0.026 *	1.001 (1.000, 1.002)	0.102	1.001 (1.000, 1.002)	0.034 *
Phosphorus (mg)	0.998 (0.996, 0.999)	0.002 **	0.999 (0.997, 1.000)	0.144	0.997 (0.996, 0.999)	<0.001 ***
Magnesium (mg)	0.999 (0.995, 1.003)	0.619	1.000 (0.996, 1.005)	0.832	1.000 (0.995, 1.005)	0.894
Iron (mg)	0.972 (0.913, 1.036)	0.372	1.014 (0.947, 1.085)	0.683	0.969 (0.893, 1.053)	0.451
Zinc (mg)	1.015 (0.995, 1.079)	0.624	0.886 (0.787, 0.999)	0.048 *	1.099 (0.990, 1.220)	0.074
Copper (mg)	1.450 (0.796, 2.642)	0.218	1.042 (0.404, 2.687)	0.931	2.754 (1.313, 5.778)	0.009 **
Sodium (mg)	1.000 (1.000, 1.000)	0.194	1.000 (1.000, 1.001)	0.487	1.000 (1.000, 1.001)	0.042 *
Potassium (mg)	1.000 (0.999, 1.000)	0.607	1.000 (1.000, 1.001)	0.516	0.999 (0.999, 1.000)	0.032 *
Selenium (μg)	1.002 (0.993, 1.012)	0.625	0.997 (0.985, 1.010)	0.674	1.000 (0.989, 1.011)	0.969
Caffeine (mg)	1.002 (1.001, 1.003)	0.003 **	1.000 (0.998, 1.002)	0.861	0.999 (0.996, 1.001)	0.297
Alcohol (g)	1.011 (0.996, 1.027)	0.146	1.013 (0.994, 1.031)	0.170	0.971 (0.950, 0.992)	0.009 **

* *p* < 0.05, ** *p* < 0.01, *** *p* < 0.001.

## Data Availability

The data supporting the findings of this study are publicly available from the NHANES. (https://www.cdc.gov/nchs/nhanes/index.htm). Accessed on 4 January 2022.
